# Impact of Long-term Glycosylated Hemoglobin in Patients with Acute Myocardial Infarction: a retrospective cohort study

**DOI:** 10.1038/s41598-020-63802-1

**Published:** 2020-04-21

**Authors:** Wonjae Lee, Sun-Hwa Kim, Chang-Hwan Yoon, Jung-Won Suh, Young-Seok Cho, Tae-Jin Youn, In-Ho Chae

**Affiliations:** Division of Cardiology, Department of Internal Medicine, College of Medicine, Seoul National University and Cardiovascular Center, Seoul National University Bundang Hospital, Seongnam-si, Gyeonggi-do Korea

**Keywords:** Cardiology, Diseases

## Abstract

Little clinical evidence supports the strict implementation of glycemic control for diabetic patients with AMI. We aimed to demonstrate the effect of long-term glycemic control on mortality in patients with diabetes mellitus after acute myocardial infarction (AMI). Eight hundred and twenty-four consecutive diabetic patients were divided into three groups according to the mean hemoglobin (HbA1c) value: <6% (group A), ≥6% to <7.5% (group B), and ≥7.5% (group C). The best long-term mortality outcome was observed in Group B, followed by groups C and A. Groups B and C were further compared in-depth because the baseline characteristics of group A differed significantly. A Cox regression analysis indicated that Group C was associated with an adjusted hazard ratio (HR) of 1.55 [95% confidence interval (CI): 1.02–2.34, P = 0.038]. An inverse probability of treatment weight analysis was performed to compare groups B and C. Group C had significantly higher mortality, compared to group B (adjusted HR: 1.58; 95% CI: 1.21–2.06, P  < 0.001). In conclusion, Glycemic status was associated with the long-term survival outcome in diabetic patients after AMI. However, further study is needed to prove whether HbA1c-targeted glycemic control can effectively improve survival after AMI.

## Introduction

A significant proportion of patients with acute myocardial infarction (AMI) have diabetes mellitus or pre-diabetes at the time of the diagnosis, and these patients have worse clinical outcome^1–3^. Higher mortality was observed in patients with AMI who presented with hyperglycemia or a higher glycated hemoglobin (HbA1c) level at admission^4,5^. Therefore, the glycemic status should be evaluated in all AMI patients, regardless whether the case involves a known history of diabetes or hyperglycemia at admission^6^. During the acute phase post-MI, current recommendations based on a study of intensive insulin therapy in critically ill patients suggest that patients should maintain a blood glucose concentration ≤11.0 mmol/L or ≤200 mg/dL but absolutely avoid hypoglycemia^7^.

However, no study has assessed the long-term glycemic control status after a diagnosed AMI and the prognostic impact of this parameter. One cohort study of patients with ST-eleveation MI (STEMI) who underwent percutaneous coronary intervention (PCI) reported long-term prognosis, but based on the HbA1c level at admission^8^.

To date, robust data have been unavailable to guide optimal glucose management (e.g., treatment thresholds and glucose targets) in patients with AMI after discharge and during follow-up. Therefore, we investigated the association between the average HbA1c level during a long-term follow-up and the all-cause mortality rate in patients with diabetes and AMI.

## Methods

### Study population and data collection

**Figure 1:**
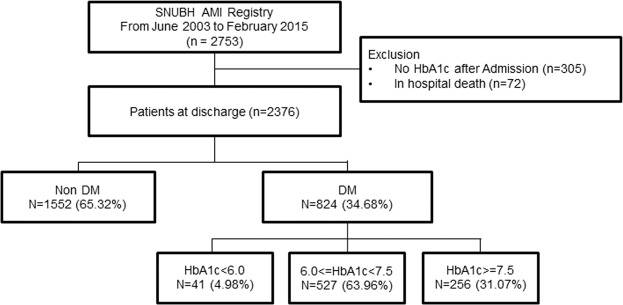
Flow chart of the establishment of the study population. A total of 824 patients with diabetes mellitus and acute myocardial infarction were analyzed.

A total of 2,753 patients with AMI were treated at our institution between June 2003 and February 2015 (Fig. 1). Among these patients, we used the Clinical Data Warehouse system to identify 2,376 patients for whom HbA1c measurements were recorded during the admission for AMI and after discharge. We identified 824 (34.7%) diabetes patients from the cohort. Other clinical variables were collected from the review of electronic medical records and the Clinical Data Warehouse system. Clinical outcomes were all cause death, non-fatal MI, stroke, and admission for heart failure. All-cause mortality, which was cross-checked using data from SNUBH AMI registry and data obtained from the Korean Ministry of Security and Public Administration to ensure no losses to follow-up. All patients were followed up from admission till the end of follow-up (the date of death or the last data acquisition from the Korean Ministry of Security and Public Administration). Therefore, each patient would have different durations of follow up. The median follow-up duration of our AMI registry was 1,962 days (interquartile range: 1,298–2,746 days). The study protocol was approved by the Institutional Review Board of Seoul National University Bundang Hospital (IRB number: B-1708-412-109). Because of the retrospective nature of the study, the requirement for informed consent from individual patients was waived.

### Definition of Diabetes and AMI

Patients with diabetes were identified using any of the following three criteria: a specified diagnosis in the medical record, a prescription record for diabetes medication, and a HbA1c level ≥ 6.5% during admission. When patients fell into former two criteria, patient was designated as known diabetes. HbA1c level ≥ 6.5% was used as a criterion only when other two criteria were absent, and patients was designated as new diabetes. Patient enrollment criteria for SNUBH AMI Registry followed the Universal Definition of Myocardial Infarction which was revised over time, and periprocedural MI was excluded in our registry.

### Statistics

For analyses of baseline characteristics such as demographic parameters, clinical variables, and procedure-related factors, Student’s unpaired-t test and the Wilcoxon rank-sum test were applied to continuous variables, which were expressed as means ± standard deviations (SD) as needed; the χ^2^ test or Fisher’s exact test was applied to categorical variables, which were presented as frequencies and percentages. To compare HbA1c levels over time between the three groups, the 6-month average HbA1c value was calculated for each patient at each time point, and the average of each group was determined and used to compare inter-group differences. A restricted cubic spline was used to visualize the relationship between the continuous HbA1c level and the relative risk of mortality. We performed survival analysis with right-censoring at 5 years to estimate the 5-year hazard of the glycemic status on mortality. The Kaplan–Meier method and log-rank test were used to construct the curves as a visual representation of differences in the primary endpoint and to demonstrate statistical significance, respectively. A Cox proportional hazards regression model was used to confirm the impact of the average HbA1c level on all-cause mortality. Covariates were HbA1c, age, male sex, body mass index (BMI), hypertension, dyslipidemia, STEMI diagnosis, previous cardiovascular disease (CVD), use of medication (beta-blockers, renin-angiotensin system (RAS) inhibitors, oral hypoglycemics, and insulin), creatinine level, left ventricular ejection fraction (cut-off value of 40%), newly diagnosed DM, and GRACE score. Variables identified as statistically significant in the univariate analysis were included in the multivariate analysis. Multicollinearity was evaluated and covariates for the adjustment were selected using the stepwise Akaike information criterion (AIC) method. The proportional hazards assumption was tested based on the scaled Schoenfeld residuals. Given the possibility of significant differences in patients’ characteristics between the groups consequent to a treatment strategy bias, we used inverse probability of treatment weighting (IPTW) to rigorously adjust for significant differences in baseline characteristics. All reported P values were 2-tailed, and a P value ≤0.05 was considered statistically significant. All statistical analyses were performed using the R Statistical Software environment (version 3.4.0; The R foundation for Statistical Computing, Vienna, Austria). All analyses were performed and verified by a professional statistician.

## Results

### Patients’ characteristics, glycemic control, and all-cause mortality during follow-up

**Figure 2:**
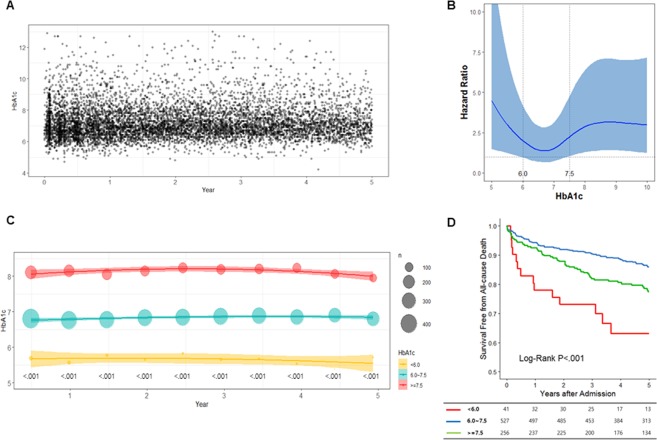
Glycated hemoglobin (HbA1c) levels after acute myocardial infarction and the association of this variable with prognosis. HbA1c was measured continuously throughout the follow-up period (**A**). The adjusted hazard ratio according to the individual mean HbA1C level for each patient, illustrated by a J-shaped restricted cubic spline curve for all-cause mortality (**B**). Patients were classified into three groups according to the mean HbA1c levels: <6.0%, ≥6% to <7.5%, and ≥7.5%; each group maintained a constant HbA1c level throughout the follow-up period. The size of the circle indicates the number of patients included at each time point, and the linear lines indicate the average and standard deviation (**C**). The cumulative 5-year survival rate differed significantly between the three groups (**D**).

A total of 824 patients with diabetes were included in the study. A total of 7,921 HbA1c levels were measured regularly throughout follow-up period (Fig. 2A). One hundred eighty-six deaths occurred during the follow-up period. When we drew a restricted cubic spline curve with an adjusted hazard ratio according to the mean HbA1c level of each patient, we obtained a J-shaped curve and identified 6% and 7.5% as the best cutoff points predictive of differential outcomes (Fig. 2B). Therefore, we classified the patients into three groups according to mean HbA1c levels: <6.0% (group A), ≥ 6% to <7.5% (group B), and ≥7.5% (group C). Groups A, B, and C included 41 (5.0%), 527 (64.0%), and 256 (31.1%) patients, respectively. The baseline characteristics of the groups are described in Table 1. Overall, patients in group A had unfavorable clinical characteristics, including older age and more frequently presented with hypertension, dyslipidemia, and a previous history of cardiovascular disease. At discharge, patients in group A was less frequently prescribed P_2_Y_12_ inhibitors and statins and more frequently prescribed diuretics. Group A also contained the highest percentage of patients with a left ventricle ejection fraction (LVEF) of <40%. This group also had a significantly lower estimated glomerular filtration rate (eGFR). Furthermore, the treatment strategies differed significantly between the groups. Here, patients in Group A more frequently underwent CABG, compared to the other groups.

**Table 1:** Baseline characteristics of the patients.

	All	Group A (HbA1c < 6%)	Group B (HbA1c ≥ 6%, < 7.5%)	Group C (HbA1c ≥ 7.5%),	p value among the three groups	P value between group B vs C
Variables	824	41	527	256		
Age	64.00 ± 12.19	68.44 ± 13.06	64.79 ± 11.74	61.67 ± 12.60	<0.001	0.001
Male sex	595(72.2)	25(61.0)	378(71.7)	192(75.0)	0.163	0.379
Known Diabetes	619(75.1)	29(70.7)	366(69.4)	224(87.5)	<0.001	<0.001
Hypertension	515(62.6)	30(73.2)	346(65.8)	139(54.3)	0.003	0.002
Dyslipidemia	206(25.1)	14(34.1)	133(25.3)	59(23.1)	0.314	0.572
Previous MI					0.135	0.064
- >30 d	52(6.4)	4(9.8)	25(4.8)	23(9.1)		
- 7~30 d	15(1.8)	0(0.0)	11(2.1)	4(1.6)		
Heart failure	13(1.6)	3(7.3)	8(1.5)	2(0.8)	0.029	0.512
Stroke	74(9.0)	7(17.1)	47(8.9)	20(7.8)	0.160	0.684
Previous CVD	138(16.7)	12(29.3)	79(15.0)	47(18.4)	0.044	0.271
Smoking					0.003	0.004
- Current	317(38.8)	9(22.0)	189(36.2)	119(46.9)		
- Former	205(25.1)	12(29.3)	129(24.7)	64(25.2)		
- Never	295(36.1)	20(48.8)	204(39.1)	71(28.0)		
BMI, kg/m^2^	24.46 ± 3.53	23.89 ± 4.39	24.45 ± 3.49	24.57 ± 3.46	0.570	0.645
Heart Rate	75.58 ± 14.45	79.22 ± 15.89	75.02 ± 14.35	76.13 ± 14.36	0.147	0.315
STEMI	386(46.9)	13(31.7)	246(46.8)	127(49.6)	0.102	0.509
Multivessel disease	579(77.4)	30(78.9)	363(77.4)	186(77.2)	0.971	>.999
SBP at discharge	118.2 ± 16.53	121.59 ± 15.56	117.70 ± 16.33	118.68 ± 17.39	0.250	0.442
DBP at discharge	66.04 ± 10.3	63.61 ± 9.65	66.06 ± 10.27	66.38 ± 10.46	0.439	0.684
<Medication>						
Aspirin	820(99.5)	41(100.0)	526(99.8)	253(98.8)	0.271	0.105
P2Y12 inhibitor	792(96.1)	35(85.4)	508(96.4)	249(97.3)	0.006	0.671
Warfarin	38(4.6)	5(12.2)	22(4.2)	11(4.3)	0.085	>.999
RAS inhibitors	666(80.8)	34(82.9)	422(80.1)	210(82.0)	0.760	0.580
Diuretics	285(34.6)	24(58.5)	170(32.3)	91(35.5)	0.003	0.404
CCB	119(14.4)	11(26.8)	81(15.4)	27(10.5)	0.014	0.064
Statin	714(86.7)	26(63.4)	463(87.9)	225(87.9)	<0.001	>.999
Beta Blocker	593(72.0)	25(61.0)	389(73.8)	179(69.9)	0.144	0.289
OHA	603(73.2)	23(56.1)	398(75.5)	182(71.1)	0.017	0.215
Insulin	176(21.4)	6(14.6)	62(11.8)	108(42.2)	<0.001	<0.001
<Echocardiography>						
Mean LV EF, %	51.58 ± 11.67	47.31 ± 15.47	52.09 ± 1127	51.21 ± 11.67	0.073	0.314
LV EF < 40%	127 (15.41)	16 (39.0)	73 (13.9)	38 (14.8)	<0.001	0.792
LVEDD	49.26 ± 6.06	49.95 ± 6.34	49.20 ± 5.86	49.26 ± 6.38	0.684	0.893
LVESD	34.31 ± 7.27	35.60 ± 7.58	34.13 ± 7.14	34.49 ± 7.50	0.310	0.522
<Lab>						
Creatinine, mg/dl	1.32 ± 1.34	2.18 ± 1.79	1.29 ± 1.44	1.23 ± 0.92	<0.001	0.448
eGFR, ml/min/1.73 m^2^	72.44 ± 38.2	46.81 ± 27.72	73.28 ± 35.71	75.17 ± 43.15	<0.001	0.592
Troponin I, ng/ml	76.33 ± 123.87	88.41 ± 184.80	72.56 ± 113.15	82.16 ± 132.92	0.631	0.321
Pro-BNP, ng/ml	3506.02 ± 7951.79	6730.20 ± 11076.72	3645.47 ± 8465.62	2653.59 ± 5827.62	0.056	0.087
CK-MB, ng/ml	57.28 ± 111.38	33.02 ± 62.94	60.89 ± 119.72	53.75 ± 98.58	0.773	0.377
hsCRP, mg/dl	5.44 ± 6.92	4.98 ± 5.00	5.30 ± 6.73	5.85 ± 7.69	0.862	0.482
Cholesterol, mg/dl	167.28 ± 42.76	150.58 ± 41.27	168.96 ± 42.81	166.44 ± 42.49	0.047	0.454
HDL, mg/dl	42.22 ± 10.34	42.29 ± 12.72	42.15 ± 10.33	42.37 ± 10.03	0.842	0.780
LDL, mg/dl	100.61 ± 35.27	93.82 ± 34.65	102.25 ± 36.38	98.18 ± 32.81	0.181	0.149
Triglyceride, mg/dl	146.77 ± 119.63	117.57 ± 92.01	147.45 ± 123.26	149.51 ± 115.32	0.106	0.826
Treatment					<0.001	0.836
- None	52(6.3)	4(9.8)	35(6.6)	13(5.1)		
- Thrombolysis	28(3.4)	0(0.0)	18(3.4)	10(3.9)		
- PCI	649(78.8)	23(56.1)	419(79.5)	207(80.9)		
- CABG	95(11.5)	14(34.1)	55(10.4)	26(10.2)		
GRACE	115.38 ± 37.13	135.28 ± 41.08	115.14 ± 35.69	112.59 ± 38.56	0.009	0.372

MI, myocardial infarction; CVD, cardiovascular disease; BMI, body mass index; STEMI, ST-elevated myocardial infarction; SBP, systolic blood pressure; DBP, diastolic blood pressure; RAS, renin-angiotensin-system; CCB, calcium channel blocker; OHA, oral hypoglycemic agent; LVEF, left ventricle ejection fraction; LVEDD, left ventricle end diastolic dimension; LVESD, left ventricle end systolic dimension; estimated glomerular filtration rate; pro-BNP, pro-brain natriuretic peptide; CK-MB, creatine kinase-MB isoenzyme; hsCRP, high sensitive C-reactive protein; HDL, high density lipoprotein; LDL, low density lipoprotein; PCI, percutaneous coronary intervention; CABG, coronary artery bypass graft.

The average HbA1c values at admission were 5.94%, 7.19%, and 8.65%. The Average HbA1c values during the 5-year follow-up were 5.68 ± 0.27 in group A, 6.83 ± 0.36 in group B, and 8.39 ± 0.90 in group C (P < 0.001). Each group maintained a constant HbA1c level throughout the follow-up period (Fig. 2C). The average HbA1c value differed significantly between the groups at every 6-month interval (P < 0.001). We further analyzed 391 patients (110 in group C, 259 in group B, and 22 in group A) for whom HbA1c data were available prior to admission for AMI. Interestingly, the average HbA1c values from before to after AMI differed significantly only in group A (before, 6.40 ± 0.66 vs. after, 5.59 ± 0.19; P = 0.002); no such difference was observed in the other two groups (Supplementary Fig. 1).

The cumulative 5-year survival rate differed significantly between the three groups and was worst in group A and best in group B (Fig. 2D). As group A included a number of patients critically ill and vulnerable to hypoglycemia compared to the other two groups, only groups B and C were considered for further analysis to investigate the potential effect of the long-term HbA1c level on mortality after AMI.

### Clinical outcomes of patients with a HbA1c level ≥ 7.5% vs. 6.0–7.4%

Compared to group C, group B had a lower all-cause mortality rate during the 5-year follow-up period (log-rank test, P = 0.002). The 1-year incidence of all-cause mortality did not differ between groups B and C (5.69% vs. 7.42%, P = 0.300; Supplementary Figs. S2 and S3). However, the survival curves continued to diverge after 1 year, and the risk of all-cause mortality was significantly higher in group C than group B (P = 0.002). The event rate for composite outcome of all-cause death, non-fatal MI, stroke, and admission for heart failure over 5 years was also significant higher in group C (Supplementary Table S1).

In a univariable Cox regression analysis, a HbA1c level ≥7.5% had a deleterious effect on survival [hazard ratio (HR): 1.72; 95% confidence interval (CI): 1.21–2.45, P = 0.003; Table 2]. Age, male sex, hypertension, BMI, dyslipidemia, STEMI presentation, previous cardiovascular disease history, beta-blocker use, RAS inhibitor use, statin use, oral hypoglycemia agent use, creatinine level, and a LVEF < 40%, and GRACE score were significantly associated with all-cause mortality. In multivariable analytical model, a HbA1c value ≥7.5% was independently associated with a worse outcome (HR: 1.55, 95% CI: 1.02–2.34, P = 0.038). Other significant factors in this model include age, BMI, hypertension, dyslipidemia, previous cardiovascular disease history, RAS inhibitor use, insulin use, and GRACE score.

**Table 2:** Cox proportional hazard analysis of covariates for all-cause mortality in group B (HbA1c ≥ 6%, <7.5%) vs. C (HbA1c ≥ 7.5%).

	Univariable			Multivariable		
	HR	CI	p	HR	CI	P
HbA1c (>=7.5)	1.72	1.21–2.45	0.003	1.55	1.02–2.34	0.038
Age	1.09	1.07–1.11	<.001	1.07	1.04–1.10	<0.001
Sex (Male)	0.68	0.47–0.98	0.037	1.48	0.99–2.22	0.059
BMI, kg/m^2^	0.83	0.79–0.88	<.001	0.91	0.85–0.97	0.002
Hypertension	1.8	1.21–2.70	0.004	1.47	0.95–2.29	0.086
Dyslipidemia	0.6	0.38–0.96	0.035	0.56	0.33–0.94	0.029
STEMI	0.61	0.42–0.88	0.007			
Previous CVD	2.39	1.62–3.53	<.001	2.15	1.40–3.28	<0.001
Beta-blockers	0.55	0.38–0.78	0.001			
RAS inhibitors	0.5	0.34–0.74	<.001	0.54	0.36–0.81	0.003
Statin	0.47	0.30–0.72	<.001			
OHA	0.55	0.39–0.79	0.001			
Insulin	2.45	1.56–3.24	<.001	1.87	1.20–2.93	0.006
Creatinine, mg/dl	1.15	1.07–1.23	<.001	1.11	0.99–1.23	0.068
LVEF (<40%)	2.62	1.77–3.89	<.001	1.42	0.92–2.21	0.117
New DM	0.73	0.47–1.13	0.160	1.44	0.88–2.34	0.142
GRACE	1.02	1.02–1.03	<.001	1.01	1.00–1.02	0.005

BMI, body mass index; STEMI, ST-elevated myocardial infarction; RAS, renin–angiotensin system; CCB, calcium channel blocker; OHA, oral hypoglycemic agent; LVEF, left ventricle ejection fraction.

**Figure 3:**
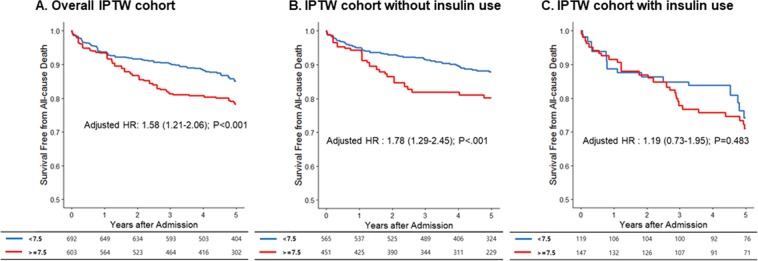
Prognosis of patients with acute myocardial infarction according to glycated hemoglobin (HbA1c) level in the inverse propensity treatment weight (IPTW) cohort. Kaplan–Meier curves were used to compare groups B (HbA1c level: ≥6% to <7.5%) and group C (≥7.5%) in the IPTW cohort. The cumulative incidence of death was significantly lower in group B.

Groups B and C differed in terms of several baseline characteristics, including age, a previous diabetes diagnosis, hypertension, smoking, and insulin use (Table 1). Therefore, we compared all-cause mortality in a propensity-matched population to determine a causal inference of long-term glycemic control on the survival outcome. The baseline characteristics of subjects in the IPTW-matched population are shown in Table 3. All clinical variables were well matched except the use of insulin, which was significantly higher in group C although this difference was markedly reduced (25.4% vs. 17.2%; P = 0.014). The cumulative survival rate was significantly higher in group B over the 5-year study period (adjusted HR: 1.58, 95% CI: 1.21–2.06; P < 0.001) (Fig. 3A). Because patients could not be matched in terms of insulin use, we separately compared the outcomes of patients without and with insulin use (Fig. 3B,C). In patients without insulin use, a HbA1C level>7.5% was significantly associated with a worse outcome (adjusted HR: 1.78, 95% CI: 1.24–2.45, P < 0.001) whereas in patients with insulin use, this factor was not significant (adjusted HR: 1.19, 95% CI: 0.73–1.95, P = 0.483).

**Table 3:** Baseline characteristics of the inverse probability of treatment weight-matched population.

	Group B	Group C	p	SD
Age	64.31 ± 11.92	63.03 ± 12.52	0.231	0.104
Sex (Male)	496 (71.67)	440 (72.95)	0.748	0.029
BMI, kg/m^2^	24.43 ± 3.51	24.40 ± 3.27	0.893	0.011
Hypertension	432 (62.39)	346 (57.34)	0.235	0.102
Dyslipidemia	170 (24.51)	140 (23.18)	0.722	0.032
STEMI	321 (46.48)	273 (45.32)	0.790	0.023
Previous CVD	105 (15.15)	107 (17.68)	0.426	0.066
Beta-blocker	512 (74.04)	419 (69.46)	0.247	0.099
RAS inhibitors	547 (79.06)	495 (82.12)	0.383	0.080
statin	613 (88.67)	544 (90.17)	0.548	0.050
P2Y12 inhibitors	667 (96.49)	582 (96.52)	0.985	0.002
Aspirin	690 (99.82)	595 (98.66)		
CCB	104 (14.97)	64 (10.62)	0.160	0.141
OHA	517 (74.75)	469 (77.81)	0.400	0.073
Insulin	119 (17.18)	153 (25.36)	0.014	0.188
Treatment			0.899	
- None	43 (6.20)	35 (5.76)		0.019
- Thrombolysis	27 (3.89)	26 (4.38)		0.024
- PCI	541 (78.25)	483 (80.16)		0.048
- CABG	81 (11.65)	59 (9.70)		0.066
Creatinine, mg/dl	1.30 ± 1.46	1.21 ± 1.03	0.399	0.072
LVEF (<40%)	102 (14.78)	79 (13.02)	0.557	0.052
New DM	190 (27.46)	120 (19.97)	0.078	0.187
GRACE	114.67 ± 35.86	113.34 ± 37.35	0.672	0.036

BMI, body mass index; STEMI, ST-elevated myocardial infarction; CVD, cardiovascular disease; RAS, renin-angiotensin-system; CCB, calcium channel blocker; OHA, oral hypoglycemic agent; PCI, percutaneous coronary intervention; CABG, coronary artery bypass graft; LVEF, left ventricle ejection fraction.

### Subgroup analysis

**Figure 4:**
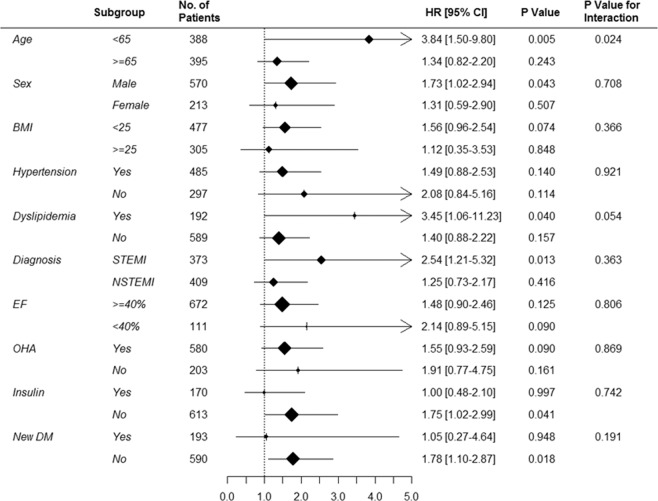
Subgroup analysis. A comparison of unadjusted hazard ratios of all-cause mortality revealed an unfavorable effect of a glycated hemoglobin (HbA1c) level >7.5% in most subgroup analyses.

To conduct a robust evaluation of the impact of the long-term HbA1c level on survival, we conducted a subgroup analysis of the entire patient population (Fig. 4 and Supplementary Fig. S4). The patients were stratified into two subgroups each according to age, sex, BMI, hypertension, dyslipidemia, type of AMI, LVEF, oral hypoglycemic agent use, insulin use, and newly diagnosed diabetes. A HbA1c level>7.5% had a consistently negative effect in all subgroup comparisons without any interaction.

## Discussion

We report for the first time an association between the long-term glycemic status with long-term all-cause mortality outcomes in diabetes patients after AMI. By stratifying patients into three groups according to the mean follow-up HbA1c level, we noticed significant differences in all-cause mortality between the three groups; here, patients with a HbA1c value ≥6% to <7.5% had the best prognosis compared to those with HbA1c values <6% or >7.5%. Furthermore, both a multivariable Cox regression model and propensity matched population analysis adjusted for baseline differences in covariates demonstrated the independently significant and deleterious effect of a HbA1c level >7.5%, compared to a level of ≥6% to <7.5%.

Pintos *et al*. reported a U-shaped association of the glucose level at admission with death or MI among STEMI patients^9^. We found that many patients with a HbA1c level <6% had a worse clinical profile, compared to those in other groups, and the average HbA1c levels both before and after AMI suggested that the reduced HbA1c levels in that group may be attributable to other causes such as malnutrition or hepatic/renal disease, rather than intensive therapy. Kosiborod *et al*. similarly reported that patients with hypoglycemia were older and had higher levels of comorbidity and that increased mortality correlated only with spontaneous hypoglycemia, but not with insulin therapy^10^. Although limited by the smaller number of patients with a HbA1c level <6% in our study, we observed a J-shaped curve for the adjusted relative risk of all-cause mortality according to the HbA1c level. We believe that patients with a consistent HbA1c level of <6% comprise a vulnerable population in which the lower glycemic level can be attributed to other causes. We therefore suggest that the use of a binary cut-off point with which to compare the effects of glycemic control may not be appropriate because the lower group may contain a subset of patients receiving intensive therapy and could thus obscure the effects of good glycemic control.

Several studies suggested that hyperglycemia has a detrimental effect on the outcomes of patients with AMI, and glycemic control may be beneficial^11–14^. However, all the above studies evaluated only the short-term glycemic status (mainly the glucose level at admission) and focused only on short-term outcomes such as in-hospital mortality. To date, only the DIGAMI (diabetes mellitus and acute myocardial infarction) trials have evaluated the effects of a relatively longer-term glycemic status. In the DIGAMI trial, diabetic patients with AMI were randomized to receive either insulin therapy or standard treatment^15^. Glycemic control status was followed until 1 year, during which the insulin therapy group exhibited a significant reduction in HbA1c levels and exhibited significantly lower mortality. However, the DIGAMI-2 trial, which randomized patients into three groups according to the intensity of the glycemic control strategy and implemented a longer follow-up period, failed to demonstrate a benefit of intensive therapy because the results of glycemic control did not differ between the three groups^16^.

There is a poor association between HbA1c and rate of hypoglycemic episodes or glycemic variability. In our study, there was a tendency that the incidence rate of hypoglycemia was higher in group C compared group B (4.71% [3.41–6.34] vs. 3.53% [2.76–4.45]). Moreover, glycemic variability inferred from the standard deviation of all blood glucose and HbA1c of patients were higher in group C (Supplementary Table S2). Although weak, there may be a relationship between mean HbA1c and rate of hypoglycemic episodes or glycemic variability, thus affecting worse clinical outcomes in group C.

Although our study featured a retrospective observational design, we attempted to determine a causal inference from the data by using a propensity score matching analysis. Our study is the first to present an association between the long-term glycemic status and long-term prognosis in diabetic patients with AMI. Our study differed from previous studies in several aspects. First, we grouped patients by the average of each individual patient’s follow-up HbA1c levels, rather than the HbA1c level at admission. Second, the inter-group differences in mean HbA1c values were maintained throughout our 5-year follow-up period. Third, various currently available oral hypoglycemic agents were prescribed, including insulin, sulfonylurea, and metformin, as well as dipeptidyl peptidase-4 inhibitors and pioglitazone. Fourth, since a very low HbA1c level was associated with a worse overall condition, we excluded such patients from further analysis. Finally, most AMI patients in our study underwent PCI.

Intensive glycemic control has long been believed to yield no benefit in terms of macrovascular disease in large clinical trials such as the UKPDS (UK Prospective Diabetes Study), a landmark study of newly diagnosed patients with type 2 diabetes^17–20^. However, the Kaplan–Meier curves representing a composite of adverse cardiovascular events in those trials diverged in the later stage of the follow-up period, the trend more pronounced in the the Action to Control Cardiovascular Risk in Diabetes (ACCORD) trial. Moreover, a post-trial study conducted by monitoring the UKPDS cohort for up to 10 years demonstrated the emergence of reductions in the risks of MI and death from any cause in the intensive therapy group^21^. Furthermore, a recent study of diabetic patients undergoing PCI showed that a HbA1c level <7.0% at 2 years was associated with a reduction in major cardiovascular events that was mainly attributed to revascularization^22^. However, only 20% of the patients had an AMI diagnosis at the time of the index PCI which limits the extension of the result to AMI patients.

No clear guideline addresses glycemic control in patients with AMI. The 2014 version of the ACC/AHA Guideline for Non-ST Elevated MI mentioned briefly that the blood glucose level should be maintained below 180 mg/dL while avoiding hypoglycemia during admission, regardless of the previous diabetes mellitus status^23^. However, the guideline did not make a recommendation regarding long-term glycemic control. Similarly, the 2017 ESC guideline did not make any recommendation regarding glycemic control in STEMI patients^6^. As our study demonstrated that the HR (including the 95% CI) exceeded 1.0 at a HbA1c level of 7.5%, we cautiously suggest that this value, if used as a target HbA1c level, could potentially improve the prognosis of diabetic patients after AMI. In the absence of data regarding the management of diabetes after MI, our study suggests that intensive hyperglycemic control may improve survival in these patients. Our findings should be investigated in further clinical trials to determine whether intensive anti-hyperglycemic treatment with new agents, such as dipeptidyl peptidase-4 inhibitors^24^, sodium glucose cotransporter 2 inhibitors^25^, and glucagon-like peptide agonists^26^, reduces major cardiovascular events including mortality after AMI.

### Limitations

Our study had several limitations. As this was a retrospective observational study of patients with available HbA1c data, the population and results were subject to selection bias. It is possible that individuals in each group might not have maintained a stable glycemic control status throughout the follow-up period. However, we analyzed the individual average HbA1c level of each subject and confirmed that all values fell within the range of cut-off values used for group classification. We used HbA1c as a diagnostic criterion for define new diabetes patient at admission. Since HbA1c was not a part of diagnostic criteria before 2010, applying this measure for earlier patient cohort may not be appropriate. However, our laboratory has applied National Glycohemoglobin Standardization Program (NGSP, certified and standardized to the Diabetes Control and Complications Trial reference value) for HbA1c assay since 2003. Therefore, we used the diagnostic criteria in this study throughout the study period for data comparability. We performed an IPTW analysis to balance the difference in clinical variables. However, as the HbA1c ≥ 7.5% group contained a significantly higher number of insulin users, the proportions of insulin users remained unbalanced even after IPTW. To mitigate this issue, we performed two additional analyses. First, a Cox regression analysis yielded an adjusted HR of 1.76 (95% CI: 1.26–2.48, P = 0.001) when insulin use was factored in the model. Second, a subgroup analysis of patients without insulin use also demonstrated a survival benefit of a HbA1c level between 6% and <7.5%. We did not have the exact duration of diabetes which can be an important contributor for clinical outcomes because we only had limited information on patients’ glycemic status before the admission since our institute is a referral center. Finally, we excluded in-hospital deaths from the study to minimize the potential effects of procedure-related complications on the analysis.

## Conclusion

Our study demonstrated the importance of glycemic status as a factor associated with better long-term prognosis in diabetic patients with AMI. We propose that a proper guideline for glycemic control should be determined through further prospective studies of diabetes patients with AMI.

## Supplementary information

Supplementary figures and tables.

## Data Availability

The datasets generated during and/or analysed during the current study are available from the corresponding author on reasonable request.
