# Supplementary material for: Impact of Long-term Glycosylated Hemoglobin in Patients with Acute Myocardial Infarction: a retrospective cohort study
Source: Sci Rep. 2020 Apr 21;10:6726. doi: 10.1038/s41598-020-63802-1 (PMC7174312; doi:10.1038/s41598-020-63802-1)
Supplement: Supplementary file 1 — Supplementary figures and tables. [file 41598_2020_63802_MOESM1_ESM.pdf]

Impact of Long-term Glycosylated Hemoglobin in Patients with Acute Myocardial Infarction: a retrospective cohort study

Wonjae Lee, MD, MBA[1], Sun-Hwa Kim, PhD[1], Chang-Hwan Yoon, MD, PhD[1], Jung-Won Suh, MD, PhD[1], Young-Seok Cho, MD, PhD[1], Tae-Jin Youn, MD, PhD [1], In-Ho Chae, MD, PhD[1]

[1]Division of Cardiology, Department of Internal Medicine, College of Medicine, Seoul National University and Cardiovascular Center, Seoul National University Bundang Hospital, Seongnam-si, Gyeonggi-do, Korea

Supplementary Fig. S1. Glycated hemoglobin (HbA1c) levels before and after acute myocardial infarction in the three groups stratified by HbA1C levels

A total of 391 patients (110 in group C, 259 in group B, and 22 in group A) with pre-admission HbA1c data were analyzed. The average HbA1c levels from before to after admission differed significantly only in group A.

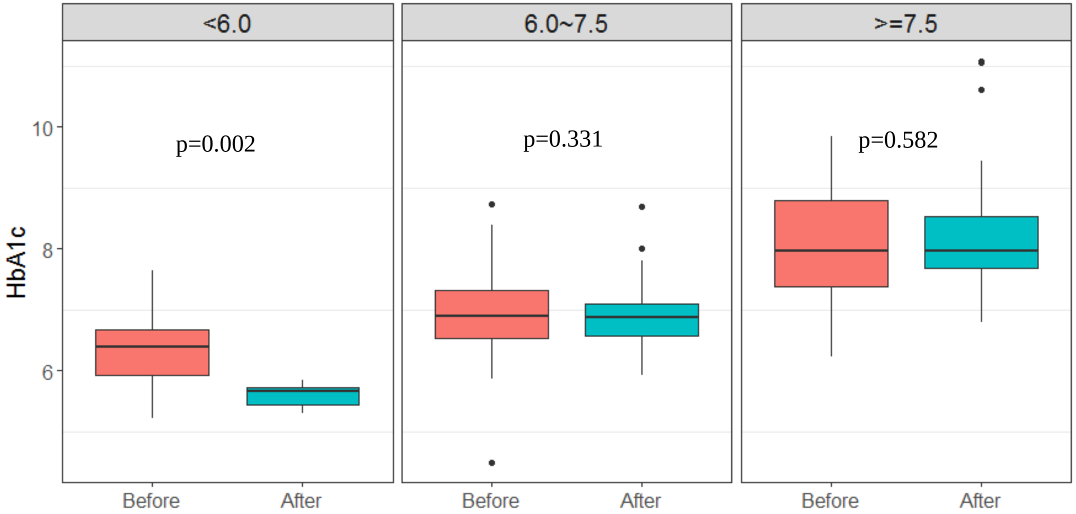

Supplementary Fig. S2. Prognosis of patients with acute myocardial infarction according to glycated hemoglobin (HbA1c) level in the original cohort: A landmark analysis at 1 year

No difference in all-cause mortality was observed at 1 year (A). However, the curves started to diverge after 1 year (B).

A. Until 1 year

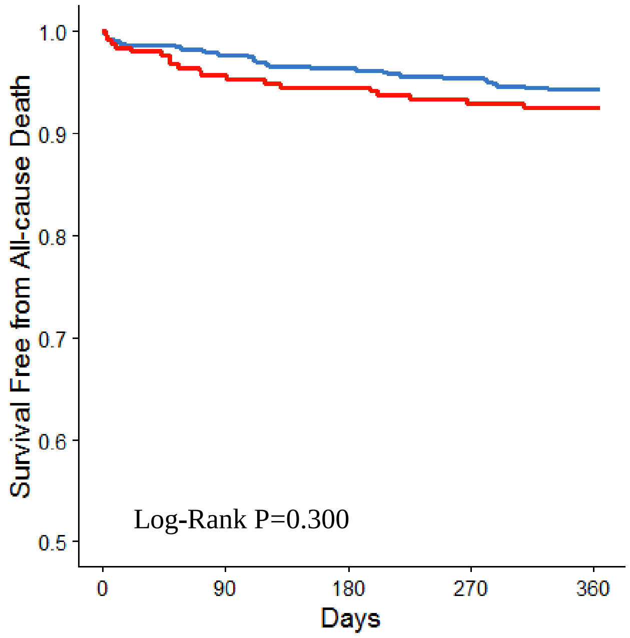

<7.5	527	515	508	503	497
>=7.5	256	245	242	238	237

B. After 1 year

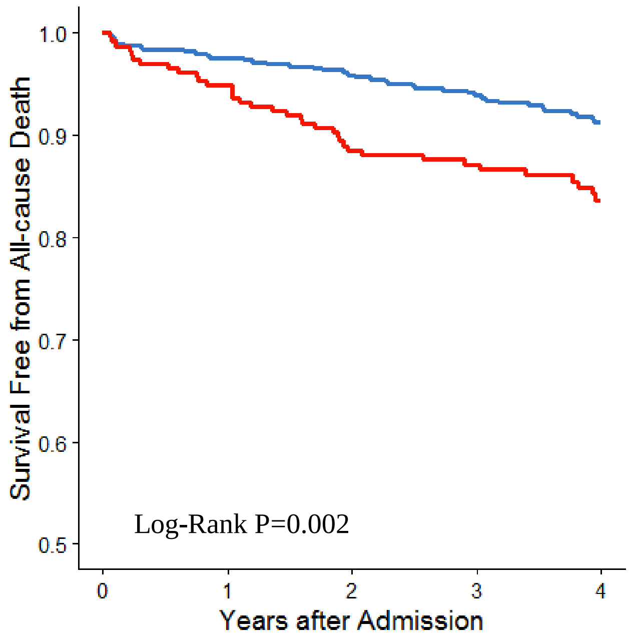

<7.5	497	485	453	384	313
>=7.5	237	225	200	176	134

Supplementary Fig. S3. Prognosis of patients with acute myocardial infarction according to the glycated hemoglobin (HbA1c) level in the inverse propensity of treatment weight (IPTW) cohort: A landmark analysis at 1 year

No difference in all-cause mortality was observed at 1 year (A). However, the curves started to diverge after 1 year (B).

A. IPTW cohort until 1 year **B. IPTW cohort after 1 year**

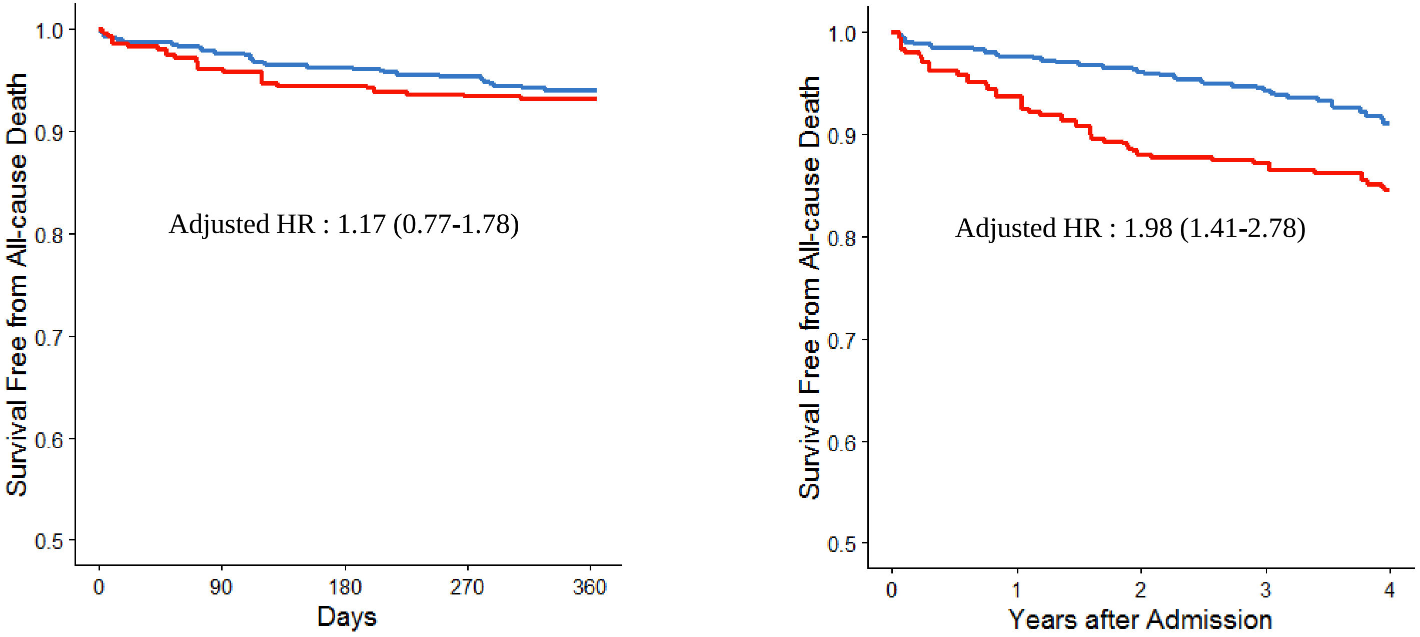

<7.5	724	708	698	691	682
>=7.5	645	620	610	603	602

<7.5	682	666	625	532	434
>=7.5	602	565	504	453	340

Supplementary Fig. S4. Prognosis of patients with acute myocardial infarction according to the HbA1c groups

A. IPTW cohort with known DM

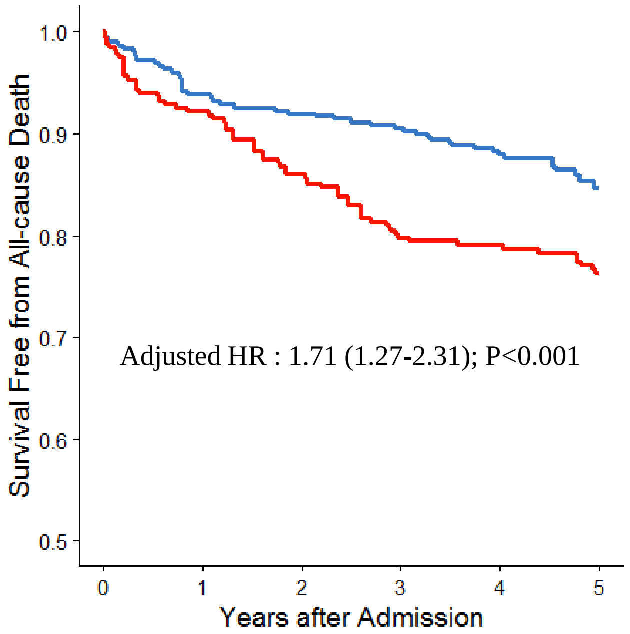

<7.5	506	476	466	435	362	286
>=7.5	464	429	400	356	313	253

B. IPTW cohort with newly diagnosed DM

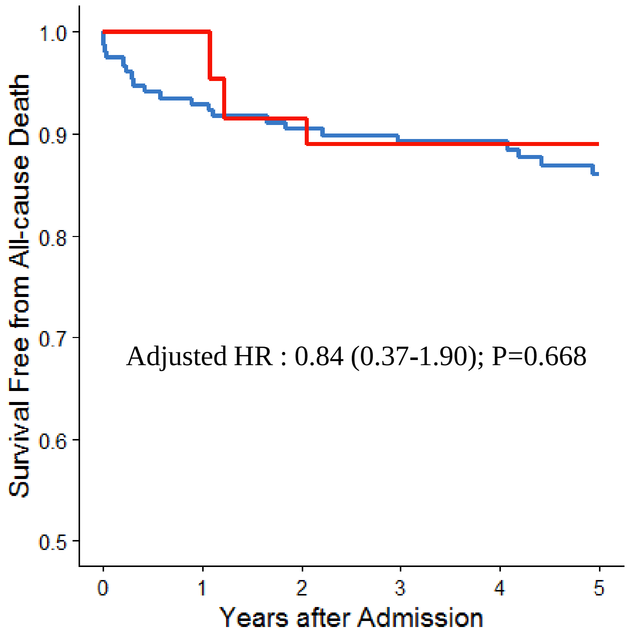

<7.5	176	164	160	150	132	108
>=7.5	69	69	63	55	53	26

Supplementary Table S1. Estimated Kaplan–Meier Events Rates Over 5 Years

	Group B	Group C	P value
All cause Death	0.14 (0.11-0.17)	0.23 (0.17-0.28)	0.002
Non-fatal Myocardial Infarction	0.04 (0.02-0.05)	0.06 (0.03-0.09)	0.100
Admission for Heart Failure	0.05 (0.03-0.07)	0.12 (0.07-0.16)	0.002
Stroke	0.04 (0.03-0.06)	0.03 (0.01-0.05)	0.400
MACE	0.18 (0.15-0.22)	0.27 (0.21-0.32)	0.009
MACE + Admission for Heart Failure	0.20 (0.17-0.24)	0.30 (0.35-0.24)	0.006

MACE, major adverse cardiovascular event

Supplementary Table S2. The glycemic variability (standard deviation) of blood glucose and HbA1c of patients

	All	Group A	Group B	Group C	P value among three groups	P value between group B vs C
**Blood glucose**						
Mean±SD	49.18±39.96	32.01±16.71	42.98±40.84	66.53±34.90	<0.001	<0.001
Mean±SE	49.18±1.59	32.01±3.05	42.98±1.99	66.53±2.59	<0.001	<0.001
**HbA1c**						
Mean±SD	0.59±0.41	0.37±0.25	0.47±0.29	0.87±0.51	<0.001	<0.001
Mean±SE	0.59±0.02	0.37±0.05	0.47±0.01	0.87±0.04	<0.001	<0.001

SD, standard deviation; SE, standard error; HbA1c, glycated hemoglobin
